# The investigation of *Helicobacter pylori* in the dental biofilm and saliva samples of children with dyspeptic complaints

**DOI:** 10.1186/s12903-017-0361-x

**Published:** 2017-03-21

**Authors:** Damla Aksit Bıcak, Serap Akyuz, Binnur Kıratlı, Merve Usta, Nafiye Urganci, Burcin Alev, Aysen Yarat, Fikrettin Sahin

**Affiliations:** 1 Faculty of Dentistry, Department of Pediatric Dentistry, Marmara University, Basibuyuk Yolu 9/3 34854 Basibuyuk, Maltepe, Istanbul, Turkey; 2 Faculty of Engineering, Department of Genetics and Bioengineering, Yeditepe University, İstanbul, Turkey; 3 Department of Pediatric Gastroenterology, Sisli Hamidiye Etfal Education and Research Hospital, İstanbul, Turkey; 4 Faculty of Dentistry, Department of Basic Medical Sciences, Biochemistry, Marmara University, İstanbul, Turkey

**Keywords:** *Helicobacter pylori*, RT-PCR, Dental biofilm, Saliva, Halitosis

## Abstract

**Background:**

The oral cavity can be an extra-gastric reservoir for *Helicobacter pylori* (*H.pylori).* This can play a role in the pathogenesis of halitosis, glossitis, recurrent aphthous stomatitis, and dental caries. The present study was conducted to detect the presence of *H.pylori* within the dental biofilm and in saliva samples collected from children suffering from dyspepsia and children without any gastrointestinal complaints. Associations with gastric infection, halitosis, and some oral parameters were also evaluated.

**Methods:**

Seventy children (aged between 5–16) with dyspepsia were selected for the study group and control group composed of 30 healthy children without dyspepsia were also included in the study. After detailed oral and clinical examinations for oral parameters, saliva, and supragingival dental biofilm samples were collected for 16S rRNA and 23S rRNA genes detection by real-time polymerase chain reaction (RT-PCR). The presence of gastric *H.pylori* was evaluated in endoscopic biopsy specimens histopathologically. Halitosis was evaluated by benzoyl-DL-arginine-naphthylamid (BANA) test. Salivary *S.mutans and Lactobacilli sp.* counts were also carried out by commercial kits.

**Results:**

*H.pylori* was histopathologically detected amongst 83% of the children with the dyspeptic condition. The detection rate of this bacteria in dental biofilm and saliva samples and halitosis were found relatively higher in the dyspeptic children rather than the control group (*p* < 0.01). Halitosis was not significantly different between dyspeptic children and those detected with *H.pylori* (*p* > 0.05). In the gastric *H.pylori* positive group with dyspepsia, DMFT/S and dmft/s numbers and plaque indices were found higher than the control group (*p* < 0.01). Only plaque indices of gastric *H.pylori* negative group with dyspepsia were found higher than the control group (*p* < 0.01). *S.mutans and Lactobacilli sp.* counts were not significantly different between gastric *H.pylori* positive and negative groups (*p* > 0.05). Comparing to those with negative for both genes, in children whose dental biofilm and saliva samples were positive for both 16S rRNA and 23S rRNA genes, significantly higher results for halitosis, and DMFS numbers and significantly lower results for dmfs numbers and pH values were found (*p* < 0.01).

**Conclusions:**

*Helicobacter pylori* can occur in the oral cavity aside and independently from the stomach. However, the high number of bacteria in the oral cavities of children with gastric *H.pylori*, an association between the presence of *H.pylori* and halitosis, DMFS, and pH were found.

**Electronic supplementary material:**

The online version of this article (doi:10.1186/s12903-017-0361-x) contains supplementary material, which is available to authorized users.

## Background

*Helicobacter pylori* (*H.pylori*) is a microaerophilic, gram-negative, spiral-shaped and motile organism which colonizes the human gastrointestinal mucosa and plays an important role in the development and recurrence of gastritis, gastroduodenal ulcer, and gastric cancer [1, 2]. This pathogenic microorganism can also be detected from both dental biofilm and saliva samples [3–5]. The oral cavity can be an extra-gastric reservoir for this bacterium and from there it travels to other sites of the body [1]. Oral presence can play a role in the pathogenesis of halitosis, glossitis, recurrent aphthous stomatitis, and dental caries [6–10].

*H.pylori* infections can occur in the early stages of life. Living in poor sanitary conditions, in a crowded family and the lack of hygienic drinking water increases the risk of contamination. The main routes of infections are oral-oral, gastro-oral (through vomiting) and faecal-oral transmission [9]. It can be vertical (parents to children) or horizontal modes of transmission (environmental contamination) [11]. Children whose mothers premasticate their food before feeding have higher rates of infection. This supports oral-oral contamination [2]. Also, individuals in close contact with domestic animals have a higher probability of acquiring this infection [12]. Gastric strains of *H.pylori* have been isolated from domestic and captive animals, birds are also a host for a wide range of Helicobacter species [13]. However, it is yet to be established as to whether *H. pylori* is only stored in the oral cavity transiently when passing to the stomach or whether the oral cavity is the original reservoir, where this bacteria can proliferate before entering, and infecting the stomach [14]. There is also a significant correlation found between infection and poor social and economic status, or high-density living conditions, food prepared in unsanitary conditions and the lack of hygienic drinking water [11, 15]. Increased prevalence rates have been reported in Chinese immigrants who use chopsticks and in African infants whose mothers premasticate their food [16, 17].

The treatment of infection usually involves administration of systemic antibiotics in combination with other drugs. Despite the current treatment regimens that lead to the successful management of *H. pylori* chronic gastritis, the reinfection rate is relatively high indicating other pathways of infection which are not affected by systemic antibiotic treatment such as dental biofilm, saliva and periodontal diseases [18]. Subgingival and supragingival plaque and saliva are found to be reservoirs for reinfection [19, 20]. This fact is due to the low concentrations of antibiotics in saliva and dental biofilm, insufficient to affect the bacteria that if local treatment is not conducted the gastric reinfection is possible [21]. *Helicobacter pylori* can also be isolated from denture fittings and hard surfaces of the oral cavity. It is not known whether their presence makes elimination more difficult during eradication therapy [22].

Bad oral hygiene is a failure factor of *H.pylori* recurrence [22, 23].

Dental biofilm provides an optimal pH, temperature and microaerophilic environment required for the survival of *H.pylori* [24]. 

The aim of this study was to detect the presence of *H.pylori* in dental biofilm and saliva samples of children suffering from dyspepsia and children without any gastrointestinal complaints and to evaluate its association with gastric infection, halitosis, and some oral parameters. The null-hypothesis is that there is no association between the presence of gastric, oral *H. pylori,* halitosis, and oral parameters.

## Methods

### Groups

One hundred children were included in this study, 47 boys and 53 girls with ages between 5–16 years (mean age = 10.22 ± 3.44 years). Seventy children with a complaint of dyspepsia who attended to the gastroenterology unit of the Sisli Hamidiye Etfal Hospital, Istanbul, Turkey, formed the study group. All these children had undergone endoscopy. Thirty children without any gastrointestinal complaints were chosen as the control group at the department of pediatric dentistry of Marmara University, Istanbul Turkey.

### Ethics

All parents of the patients and controls gave informed written consent for the participation of their children in the study, all study protocols were also approved by the Marmara University, Institute of Health Sciences Non-invasive Clinical Research Studies Ethics Committee (21.12.2012-13).

### Inclusion criteria

The study group consisted of children willing to give consent, having dyspeptic complaints who decided to undergo endoscopy. The control group consisted of children willing to give consent, not having dyspeptic complaints, all of whom were healthy individuals without systemic diseases and not having previous and present gastrointestinal complaints or disorders [4, 24–27].

### Exclusion criteria

Exclusion criteria for the study and control groups were the use of nonsteroidal anti-inflammatory drugs (NSAIDs), proton pump inhibitors and antibiotics within 1 month prior to the clinical study, previous eradication therapy, eating, drinking and brushing teeth within previous 1 h and not being a volunteer [4, 24–27].

### Collection of gastric specimens and histopathologic examination

Endoscopy was performed with a gastro-duodenoscope (Olympus® GIF-XP 150 N, Tokyo, Japan) on 70 patients attending the gastroenterology unit of the Sisli Hamidiye Etfal Hospital. Biopsy forceps were sterilized and endoscopes were fully disinfected before and after each examination. During endoscopy, 2 biopsies were taken from the esophagus, gastric antrum, and corpus. Among patients undergoing endoscopy, macroscopic findings were noted, antrum, body, and duodenum biopsies were taken from all cases unexceptionally. Histological diagnosis was made in accordance with the modified Sydney system [28]. These two specimens were sent to the laboratory for histological examination. The samples were stained with hematoxylin and eosin to show the presence of bacteria histologically [4, 12, 24, 29, 30].

### Questionnaire

Medical histories and demographic data (education and socioeconomic status, having contact with pets, total population living at home) were recorded using a questionnaire (Additional file 1). The questionnaire was validated statistically and the minimum sample size was detected with power analysis. The questionnaire was created according to previous studies [1–30]. A detailed medical history was taken from all volunteers. In addition, a number of questions concerned the presence of disease, regularly used drugs, the presence of dyspeptic symptoms, including epigastric pain, nausea, diarrhea, constipation, reflux, vomiting, and regurgitation were asked to the parents of the children participated to this study. [12, 16, 17, 31–33].

### Oral examination

All children were clinically examined in order to assess their dental status. The clinical measurements were recorded by one examiner. Oral examinations were conducted under a portable light using a CPI (community periodontal index) explorer and a disposable mirror, with a cotton swab to dry the teeth. The diagnostic criteria followed the WHO protocol [34]. Oral examination involved assessment of dentition (number of teeth, carious teeth, plaque index and gingival index), halitosis and hygienic procedures (frequency of daily toothbrushing, sort of used toothbrush, a yearly frequency of changing toothbrush, feeding with premasticated food, common usage of glasses, spoons in a family) [25, 35–42].

**Dental caries** were diagnosed at the tooth surface level according to the WHO 1997 criteria [43]. To determine the DMFT/dmft indices, the total numbers of decayed, missing, and filled teeth were calculated.

**Plaque index** was evaluated according to Silness and Löe plaque index, and scored according to four grades (0: No plaque, 1: A film of plaque adhering to the free gingival margin and adjacent area of the tooth, which can not be seen with the naked eye, 2: Moderate accumulation of plaque within the gingival pocket, on the gingival margin and/or adjacent tooth surface, which can be seen with the naked eye, 3: Abundance of soft matter within the gingival pocket and/or on the tooth and gingival margin) [44].

**The gingival index** was evaluated according to Löe and Silness and scored according to four grades (0: No inflammation, 1: Mild inflammation, slight change in color, slight edema, no bleeding on probing, 2: Moderate inflammation, moderate glazing, redness, bleeding on probing, 3: Severe inflammation, marked redness and hypertrophy, ulceration, tendency to spontaneous bleeding [45].

The plaque and gingival index scores for four regions of every tooth were recorded and then calculated to get the mean score for each child. The scores from the four areas of the tooth were added and divided by four in order to give the plaque and gingival index for the tooth as numeric form.

**Halitosis** was evaluated by using organoleptic scoring and the benzoyl-DL-arginine-naphthylamid (BANA) test. Organoleptic scoring was done according to Rosenberg Scale [46] where 0: absence of odor, 1: barely noticeable odor, 2: slight malodor, 3: moderate malodor, 4: strong malodor, and 5: severe malodor. In order to detect halitosis with the BANA test (BANA test, Knowell Therapeutic Technologies Inc., Canada) a cotton tip swab was wiped on the posterior dorsum of the tongue, removing as much coating material as possible as this coated swab was wiped onto the lower reagent pad on the BANA test strip. Each step was done following the manufacturer’s protocol. The presence of at least 10^4^ cells of *Treponema denticola, Porphyromonas gingivalis*, and *Tannerella forsythia* results in a blue color on the card. The darker the blue shows more organisms present. BANA test results were given as negative (no blue color), weakly positive (faint blue color) or positive (definite blue color); comparing the upper, salmon-colored reagent pad with the sample chart on the test bottle label. Negative means no odor; weakly positive means low odor, and positive means high odor [47–54].

### Collection of dental biofilm and saliva specimens

Subjects were not allowed to clean their teeth or to eat 1 hour prior to taking a sample of dental biofilm and saliva. Supragingival dental biofilm was removed from all the tooth surfaces with a sterile curette from all children participating in this study. Air drying and sterile cotton rolls were used to avoid saliva contamination. Dental biofilm was collected by an upward scrape against the tooth surface. Approximately 2–3 ml of non-stimulated saliva was also collected in a 15 ml test tube. Both dental biofilm and saliva samples were collected from 70 dyspeptic patients immediately before endoscopy. Dental biofilm and saliva samples were frozen immediately and stored at −20 °C until required for DNA extraction [3, 12, 27, 55–57].

### Preparation of DNA from dental biofilm and saliva specimens and amplification of 16S rRNA and 23S rRNA genes

DNA Extraction: From all dental biofilm and saliva specimens, DNA was extracted with the QIAamp DNA Mini kit (Qiagen GmbH, Hilden, Germany) according to the manufacturer’s recommendations [3, 58]. Briefly, 20 μl of a proteinase K solution was added to the QIAamp Mini Spin Columns (Qiagen GmbH, Hilden, Germany) with an Eppendorf Research plus pipette, Eppendorf AG, Germany), than 200 μl of each dental biofilm and saliva samples and 200 μl of AL buffer added and incubated at 56 °C (OLS200 Shaking bath- Grant Scientific, UK) and centrifuged (Micro 22R Centrifuge, Hettich, Germany) until the sample material was dissolved completely. Following several centrifugation and wash steps, the purified template DNA was eluted with 200 μl of AE buffer. 2.5 μl of the DNA eluate thus obtained was transferred directly to the corresponding PCR reaction mixtures. The remainder was stored at 4 °C. The quantity and concentration of the extracted DNA were assessed by means of a Nanodrop spectrophotometer. The NanoDrop 2000/2000c software program was used. (Nanodrop 2000, Thermo Fisher Scientific - NanoDrop products, USA) [3, 58].

Amplification of a 120-bp fragment of the 16S rRNA genes was performed by using primers 16S-880fw (5′-ATAGACGGGGACCCGCACAAG-3′) and 16S-999rv (5′-TGGCAAGCCAGACACTCCA-3′). Also, amplification of a 425-bp fragment of 23S rRNA genes was performed by using primers Hp23–1 (5’-CCACAGCGATGTGG TCTCAG-3’) and Hp23–2 (5’-CTCCAT AAGAGCCAAAGCCC-3’) [59–61]. The oligonucleotides were synthesized at Metabion International AG, Germany. Real-time polymerase chain reaction (RT-PCR) was carried out in a volume of 10 μl reaction mixture, containing 5 μl Power SYBR Green (Applied biosystems, UK.), 0.5 μl Primer F, 0.5 μl Primer R (Metabion International AG, Germany), 1.5 μl sterilized water and 2.5 μl of each DNA template. The PCR reaction components were added to reaction plates then sealed with optical covers. Reaction plates were loaded into the real-time PCR instrument (Bio-Rad CFX96, Applied Biosystems, UK), following the manufacturer’s instructions. Each amplification reaction cycle consisted of 95 °C for 10 min for denaturation when using genomic DNA, annealing at 95 °C for 15 sec and extension at 60 °C for 1 min. Samples were amplified for 40 consecutive cycles. CFX Manager^TM^ Software program (Bio-Rad Laboratories, Inc, UK) was used in interpreting results. Quantifications of template concentrations were calculated by comparison with a standard curve, plotted from a dilution series of 16S rRNA and 23S rRNA genes.

### Evaluation of Streptococcus mutans and Lactobacilli levels in saliva samples

*Streptococcus mutans* (*S.mutans*) was detected in saliva using Dentocult SM kit (Orion Diagnostica, Espoo, Finland). The analysis was carried out after incubating the test strip in saliva by visually evaluating the colony density according to the manufacturer’s instructions. (Class 1: <10^5^ bacteria/ml saliva, Class 2: 10^5^ – 10^6^ bacteria/ml saliva, Class 3: > 10^6^ bacteria/ml saliva). The same procedure was employed to determine *Lactobacilli* in saliva using Dentocult LB stripes Low: <10^3^ bacteria/ml saliva, Moderate: 10^3^ – 10^4^ bacteria/ml saliva, High: >10^4^ bacteria/ml saliva). (Orion Diagnostica, Espoo, Finland) [57, 62].

### Evaluation of salivary flow rate, pH, and buffering capacity

After chewing paraffin wax gums, the children were requested to stir for 5 minutes in order to detect stimulated salivary flow rates. Salivary pH was measured with the pH meter (Thermo Scientific™ Orion™ 3-Star Benchtop pH Meter, Thermo Fisher Scientific Inc. USA) and the buffering capacity was measured using Ericsson method from all subjects participated in this study [63, 64]. All the tests in the study were carried out by a specialist.

### Statistical analysis

IBM SPSS Statistics 22 program was used for the statistical analysis of this study. The normal distribution of these parameters was evaluated by the Shapiro-Wilk test. In addition, the Oneway ANOVA test and the Tukey HDS test were used for the comparison of quantitative datas with descriptive statistical methods (mean, standard deviation, frequency) as well as between normal distribution parameters. The Kruskal-Wallis test was used in the comparison of the groups with no normal distribution and the Mann–Whitney-U test was used to determine the group causing the difference. Student t test was used for the comparison of the normal distribution of two groups, and Mann–Whitney-U test was used for comparison of two groups of non-normal distribution. Chi-square test, Fisher's Exact Chi-square test and Continuity (Yates) correction were used for comparison of qualitative datas. Significance was assessed at *p* <0.05 level. Multivariate backward logistic regression models were used to calculate crude and adjusted odds ratios with 95% confidence intervals (*p* < 0.05).

## Results

### Detection rates of *H.pylori* in gastric, dental biofilm and saliva samples

*H. pylori* were found in gastric biopsies of 82.9% (58/70) among the dyspeptic children who underwent endoscopy. The detection rate of this organism in dental biofilm and saliva samples with the amplification of both 16S rRNA and 23S rRNA genes are displayed in Table 1. It is clear that the detection rate increased when one gene amplification was used and decreased when two genes were amplified for bacterial identification. Thus in the evaluation of the oral findings; dental biofilm and saliva samples were accepted as *H.pylori* positive if both genes were detected.

**Table 1:** The detection rate of 16S rRNA, 23S rRNA and 16S + 23S rRNA in dental biofilm and saliva samples, halitosis (BANA test) results and oral findings among study and control groups

		Dyspeptic children who underwent endoscopy (Study group) (*n* = 70)		Children without dyspepsia (Control Group) (*n* = 30)	*P*
Endoscopic Examination Results		Gastric *H.pylori* (−) (*n* = 12, 17%)	Gastric *H.pylori* (+) (*n* = 58, 83%)	No endoscopic examination	
Saliva	16 S RNA (+)	10 (83.3%)	46 (79.3%)	14 (46.7%)^*^	^a^0.004
	23 S RNA (+)	10 (83.3%)	55 (94.8%)	25 (83.3%)	^a^0.167
	16 S RNA+ 23 S RNA (+)	9 (75.0%)	44 (75.9%)	12 (40.0%)^*^	^a^0.003
Dental Biofilm	16 S RNA (+)	10 (83.3%)	49 (84.5%)	30 (100%)	^a^0.070
	23 S RNA (+)	10 (83.3%)	53 (91.4%)	5 (16.7%)^*^	^a^0.001
	16 S RNA+ 23 S RNA (+)	8 (66.7%)	45 (77.6%)	5 (16.7%)^*^	^a^0.001
Halitosis (BANA Test Results)	Negative	(*n* = 1, 8.3%)	(*n* = 4, 6.9%)	(*n* = 12, 40%)	^a^0.001
	Weak Positive	(*n* = 5, 41.7%)	(*n* = 34, 58.6%)	(*n* = 14, 46.7%)	
	Positive	(*n* = 6, 50%)	(*n* = 20, 34.5%)	(*n* = 4, 13.3%)^*^	
Oral Findings	DMFT	4.5 ± 4.86	5.31 ± 3.99^**^	2.19 ± 2.14	^b^0.001
	DMFS	7.1 ± 4.7	7.62 ± 7.7^**^	2.27 ± 2.16	^b^0.001
	dmft	4.67 ± 2.58	3 ± 2.79^**^	7.41 ± 4.68	^b^0.001
	dmfs	9.5 ± 8.57	5.32 ± 5.38^**^	15.59 ± 10.91	^b^0.001
	Gingival Index	0.42 ± 0.77	0.27 ± 0.49	0.04 ± 0.09	^b^0.113
	Plaque Index	0.63 ± 0.36^**^	0.78 ± 0.62^**^	0.34 ± 0,3	^b^0.001
	Salivary Flow Rate	5.04 ± 2.17	6.55 ± 2.83	5.85 ± 2.17	^c^0.139
	Saliva pH	7.4 ± 0.32	7.45 ± 0.34	7.61 ± 0.38	^c^0.096

^a^Chi-square test ^*^ *p* < 0.01: significantly different from both dyspeptic groups ;^b^Kruskal Wallis test, ^c^Oneway ANOVA test, Mann Whitney U: ^**^ *p* < 0.01: significantly different from control group

BANA test results: negative (no blue color, no odor), weakly positive (faint blue color, low odor) or positive (definite blue color, high odor)

DMFT/S: decayed, missing and filled permanent teeth/surfaces; dmft/s: decayed, missing, and filled primary teeth/surfaces

16S + 23S rRNA: 16S and 23S ribosomal RNA genes of *H.pylori*

In dental biofilm and saliva samples, 16S rRNA and 23S rRNA genes were found relatively higher in dyspeptic children in comparison to the control group (*p* < 0.01). There were no differences found between gastric *H.pylori* positive and negative groups (Table 1).

**Fig. 1:**
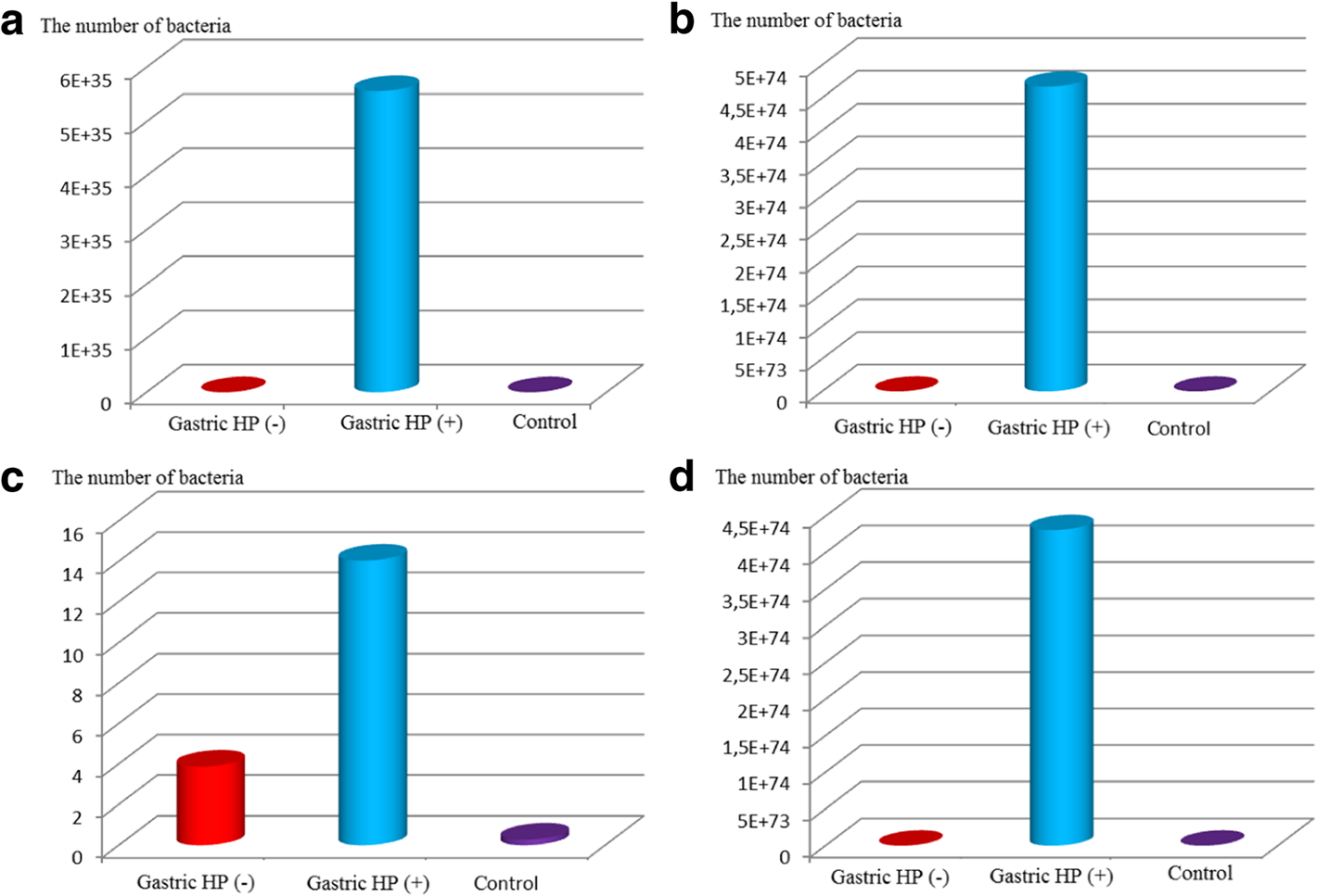
**(a)** Real-time PCR quantitation of 16S rRNA gene in dental biofilm, **(b)** Real- time PCR quantitation of 23S rRNA gene in dental biofilm, **(c)** Real-time PCR quantitation of 16S rRNA gene in saliva, **(d)** Real- time PCR quantitation of 23S r RNA gene in saliva

The quantity of *H.pylori* detected in the dental biofilm of gastric *H.pylori* positive group was found significantly higher than the negative and control groups with the amplification of 16S rRNA and 23S rRNA genes (Fig. 1a, Fig. 1b). The quantity of the organism detected in saliva of gastric *H.pylori* positive and negative groups was found significantly higher than the control group with the amplification of 16S rRNA gene (Fig. 1c). Based on 23S rRNA gene amplification the quantity detected in saliva of gastric *H.pylori* positive group was found significantly higher than other groups (Fig. 1d).

### Halitosis

According to the BANA test, the frequency of halitosis of dyspeptic children was found higher than the control group. Halitosis was not significantly different between gastric *H.pylori* positive and negative children (Table 1). Also, organoleptic scoring of halitosis was found to be statistically non-significant between groups (data was not shown). Halitosis by BANA test was found higher in children who were 16S + 23S rRNA (+) in the dental biofilm and saliva samples than children who were 16S + 23S rRNA (−) in oral samples which are shown in Table 2. Organoleptic scoring was also found statistically non-significant between children with 16S + 23SRNA (+) and (−) in the dental biofilm samples. Among children who were 16S + 23SRNA positive in saliva, moderate malodor was found higher than children with 16S + 23SRNA (−).

**Table 2:** Halitosis (BANA test) results and oral findings among children who were 16S + 23S rRNA positive and negative in dental biofilm and saliva

		Dental Biofilm		*p*	Saliva		*p*
		16S + 23S RNA (+) (*n* = 58) n (%)	16S + 23S RNA (−) (*n* = 42) n (%)		16S + 23S RNA (+) (*n* = 65) n (%)	16S + 23S RNA (−) (*n* = 35) n (%)	
Halitosis (BANA Test Results)	Negative	5 (8.6%)^*^	12 (28.6%)	^a^0.007	6 (9.2%)^**^	11 (31.4%)	^a^0.014
	Weak Positive	30 (51.7%)^*^	23 (54.8%)	^a^0.007	36 (55.4%)^**^	17 (48.6%)	^a^0.014
	Positive	23 (39.7%)^*^	7 (16.7%)	^a^0.007	23 (35.4%)^**^	7 (20%)	^a^0.014
		Mean ± SD	Mean ± SD		Mean ± SD	Mean ± SD	
Oral Findings	DMFT	5.22 ± 4.2^*^	3.03 ± 2.98	^b^0.005	4.72 ± 3.85	3.53 ± 3.91	^b^0.092
	DMFS	7.43 ± 7.91^*^	3.19 ± 3.06	^b^0.001	6.67 ± 7.43^*^	3.73 ± 4.38	^b^0.024
	dmft	4.1 ± 3.77	6.1 ± 4.5	^b^0.066	3.94 ± 4.13^*^	6.69 ± 3.95	^b^0.004
	dmfs	7.33 ± 7.24^*^	13.13 ± 11.05	^b^0.023	7.26 ± 8^*^	14.35 ± 10.52	^b^0.002
	Gingival Index	0.27 ± 0.49	0.15 ± 0.44	^b^0.059	0.27 ± 0.51^*^	0.12 ± 0.36	^b^0.005
	Plaque Index	0.72 ± 0.6^*^	0.51 ± 0.43	^b^0.049	0.71 ± 0.59	0.50 ± 0.42	^b^0.088
	Salivary Flow Rate	6.48 ± 2.93	5.71 ± 2.02	^c^0.146	6.48 ± 2.89	5.57 ± 1.85	^c^0.097
	Saliva pH	7.42 ± 0.32^*^	7.59 ± 0.37	^c^0.019	7.42 ± 0.31^*^	7.62 ± 0.39	^c^0.009

^a^Chi-square test ^*^ *p* < 0.01 and ^**^ *p* < 0.05 significantly different from 16S + 23SRNA (−)

^b^Mann Whitney U test ^c^Student t test SD: standard deviation

^*^ *p* < 0.01 significantly different from 16S + 23SRNA (−)

BANA test results: negative (no blue color, no odor), weakly positive (faint blue color, low odor) or positive (definite blue color, high odor)

DMFT/S: decayed, missing and filled permanent teeth/surfaces; dmft/s: decayed, missing, and filled primary teeth/surfaces

16S + 23S rRNA: 16S and 23S ribosomal RNA genes of *H.pylori*

### Oral parameters

Amongst the gastric *H.pylori* positive group, DMFT/S and dmft/s numbers were found higher than the control group (*p* < 0.01). Plaque indices of gastric *H.pylori* positive and negative groups were found higher than the control group (*p* < 0.01). The effect of variables like gingival index, salivary flow rate, pH and buffering capacity were found to be statistically not significant between gastric *H.pylori* positive, negative and control groups (Table 1). DMFT/S numbers and plaque indices of children who were 16S + 23S rRNA (+) in the dental biofilm were found higher, dmfs and pH levels were found lower than children who were *H.pylori* negative in dental biofilm (Table 2). In the multivariate logistic model (Table 3); dmfs and pH were found influential (*p* < 0.05) and decrease *H.pylori* presence in dental biofilm. dmfs [OR] = 0.904 and pH [OR] = 0.053; 95% confidence interval of dmfs [CI] = 0.826, 0.990 and pH [CI] = 0.005, 0.630.

**Table 3:** Multivariate logistic regression analysis of different variables associated with *H.pylori* (16S + 23S rRNA) in dental biofilm

		Odds Ratio	%95 CI		*p*
			Lower	Upper	
Step 1	DMFT	0.098	0.005	1.829	0.120
	DMFS	10.000	0.619	161.469	0.105
	dmfs	0.906	0.828	0.992	0.033
	Plaque Index	1.505	0.421	5.374	0.529
	pH	0.055	0.005	0.651	0.021*
Step 2	DMFT	1.007	0.005	1.671	0.105
	DMFS	11.294	0.684	186.402	0.090
	dmfs	0.904	0.826	0.990	0.029*
	pH	0.053	0.005	0.630	0.020*

*p < 0.05 Nagelkerke R^2^ : 0.372 (%71.1)

DMFT/S: decayed, missing and filled permanent teeth/surfaces; dmft/s: decayed, missing, and filled primary teeth/surfaces

In the multivariate logistic model DMFT, DMFS, dmfs, plaque index and pH parameters were found influential to *H.pylori* (16S + 23S rRNA) presence in dental biofilm

DMFS numbers and gingival indices of children who were 16S + 23S rRNA (+) in the saliva were found higher, pH levels and dmft/s numbers were found lower than those who were *H.pylori* negative in saliva samples. In the multivariate logistic model (Table 4); dmfs was found influential (*p* < 0.05) and decrease *H.pylori* presence in saliva [OR] = 0.864, 95%[CI] = 0.785, 0.950.

**Table 4:** Multivariate logistic regression analysis of different variables associated with *H.pylori* (16S + 23S rRNA) in saliva

		Odds Ratio	%95 CI		*p*
			Lower	Upper	
Step 1	DMFS	1.283	0.929	1.772	0.130
	dmft	1.023	0.679	1.540	0.913
	dmfs	0.869	0.715	1.056	0.157
	Gingival Index	2.176	0.231	20.507	0.497
	pH	0.492	0.047	5.114	0.552
Step 2	DMFS	1.279	0.934	1.751	0.125
	dmfs	0.877	0.794	0.968	0.009*
	Gingival Index	2.173	0.230	20.520	0.498
	pH	0.488	0.047	5.035	0.547
Step 3	DMFS	1.270	0.933	1.730	0.129
	dmfs	0.872	0.790	0.963	0.007*
	Gingival Index	1.868	0.204	17.150	0.581
Step 4	DMFS	1.282	0.942	1.744	0.114
	dmfs	0.864	0.785	0.950	0.003*

*p < 0.05 Nagelkerke R^2^: 0.384 (%71.2)

DMFT/S: decayed, missing and filled permanent teeth/surfaces; dmft/s: decayed, missing, and filled primary teeth/surfaces

In the multivariate logistic model; DMFS, dmft/s, gingival index and pH parameters were also found influential to *H.pylori* (16S + 23S rRNA) presence in saliva

Frequency of daily toothbrushing, the duration of toothbrushing, yearly frequency of changing a toothbrush, the sort of toothbrush used (soft or hard), toothpaste, mouth rinse and dental floss usage, feeding with premasticated food, common usage of glasses and spoons amongst family members, daily sugar intake and drinking habits, *S. Mutans and Lactobacilli sp*. numbers did not differ significantly between gastric *H.pylori* positive, negative and control groups. Also, these parameters were found statistically non-significant between children who were 16S + 23S rRNA positive and negative in oral samples (data not shown in the table).

### Demographic parameters

The influence of certain variables like family income, family education, and population status and the presence of pets in the household did not significantly contribute to the gastric *H.pylori* positive, negative and control groups (data not shown in the table).

## Discussion

### Detection rate of gastric *H.pylori* histopathologically

The detection rate of gastric *H.pylori* was found as 82.9% among dyspeptic children in the current study. Medina et al. [4], Malik et al. [65] and Jafri et al. [66] reported the detection rate as 88.4%, 51%,53% among dyspeptic children respectively. The result of our study was in accordance with the result of Medina et al. [4] but found higher than Malik et al. [65] and Jafri et al. [66]. Detection rate varies when studying different populations and age groups. In developing countries, it was shown that the detection rate was higher than in developed countries [67]. The occurrence of gastric infection reaches 80% and 40% in developing and developed countries respectively [68].

### Detection rates of oral *H.pylori* with RT-PCR

In studies in which *H.pylori* was detected in dental biofilm and saliva with RT-PCR, Burgers et al. [3], Altındiş et al. [55], Sayed et al. [69] and Martinez-Gomis et al. [70] used adults as the subjects, Bharath et al. [71] completed their studies using with adults and children. Investigations conducted by, Valdez-Gonzales et al. [5] and Ou et al. [72] involved the use of only children. Thus, it was very hard to compare the detection rate and quantity of *H.pylori* in dental biofilm and saliva samples of this study with other studies because of the differences in the methods used in the detection of *H.pylori* (CLOtest, histology, culture, PCR method), differences in studied population, patient and age groups, oral health status, used primers in PCR with different sensitivity and specificity, type and number of clinical samples collected and laboratory procedures. In our study RT- PCR method was used which has higher sensitivity and specificity in detection than other methods. Kignel et al. [73] reported that the success of RT- PCR method to detect *H. pylori* in low numbers in the oral cavity is very high. Chaudhry et al. [61] reported that the detection in dental biofilm samples is more reliable when two genes of the bacterium are simultaneously amplified as compared to one gene amplification only. They reported that with simultaneous amplification of two bacterial genes 51.6% of the dental biofilm samples were positive while this prevalence increased to 73% when only one gene amplification was used for bacterial identification. Thus, in the present study, two genes of the bacterium were amplified. Our results were found in accordance with Chaudhry et al. [61]. The detection rate increased when one gene amplification was used and decreased when two genes were amplified for bacterial identification. With the amplification of two genes; RT-PCR identified *H.pylori* in the dental biofilm and saliva samples of 8 (66.7%), 9 (75%) among gastric *H.pylori* (−) children, 45 (77.6%), 44 (75.9%) among gastric *H.pylori* (+) children and 5 (16.7%), 12 (40%) among control group respectively. The presence of *H.pylori* (independent from quantity even though in small numbers) in dental biofilm and saliva samples of gastric *H.pylori* negative children and control group showed a lack of correlation between oral and gastric bacterial colonization. Therefore *H.pylori* can be found in the oral cavity independently from a gastric infection. In accordance with our findings, Bürgers et al. [3] and Suzuki et al. [8] reported that the detection of *H.pylori* in the oral cavity can occur independently from gastric colonization suggesting that the human oral cavity could represent an important reservoir for this bacterium.

With the RT-PCR method, it is also possible to quantitatively determine the amount of bacteria. The low number of the bacteria in the oral cavity may be a problem in diagnostics and may often be overlooked, which makes the data unclear. Polymerase chain reaction assay is able to detect as few as 10 *H.pylori* cells, and it provides the most sensitive and most specific test for the detection of *H.pylori* in samples taken from sites outside the stomach [74]. In this study, the amount of detected *H.pylori* in the dental biofilm and the saliva samples of gastric *H.pylori* positive children were significantly higher than negative and control groups. With amplification of 16S rRNA and 23S rRNA genes, *H.pylori* quantity in the dental biofilm of gastric *H.pylori* positive children was found significantly higher than negative and control groups. With amplification of the 23S rRNA gene, *H.pylori* quantity in the saliva of gastric *H.pylori* positive children was found significantly higher than negative and control groups [Fig. 1]. Our results were found in accordance with Czesnikiewicz-Guzik et al. [75] and Nasr elahei et al. [76]. *Helicobacter pylori* might be a part of normal oral microenvironment without being pathogenic for the stomach, remaining in normal immunological balance with the host, or even protecting against other pathogens. In addition, the small number existing at this level might not be a real danger for the gastric infection. However, when the immune system of the host becomes impaired, a pathogenic sense is induced. The first place of entrance to the human body might be the oral cavity. Siddiq et al. [77] also implicated that oral cavity may be the first place for colonization and then the infection involves the gastric mucosa. In the present study, the quantity of *H.pylori* detected in the oral cavities of gastric *H.pylori* negative children and control group were quite low and not enough to colonize stomach. With poor oral hygiene and with the accumulation of the dental biofilm, the number of bacteria can increase in the oral cavity as a result of microaerophilic feature and be sufficient for starting a gastric infection in the stomach through swallowing. As shown in this study, a considerable amount of *H.pylori* detected in the oral cavities of gastric *H.pylori* positive children might enough to colonize the stomach by swallowing.

### Halitosis

Halitosis was also evaluated by two methods in our study. Organoleptic scoring and the BANA test were both used. According to organoleptic scoring, there was no clear relationship found between the gastric presence of *H.pylori* and halitosis. This result was in agreement with some other studies [47, 48, 59, 78]. However was not in agreement with the results of the studies conducted by Tiomny et al. [49], Serin et al. [50], Li et al. [51], Katsinelos et al. [79] and Chen et al. [80]. Loesche et al. [52] described a microbiological test denominated the BANA test, which uses a chromophore added to a synthetic peptide as a substrate. This technique has shown better results in comparison with other microbiological tests [53, 54]. According to BANA test; no clear relationship was found between the gastric presence of *H.pylori* and halitosis. The BANA test results of dyspeptic children who underwent endoscopy were found higher than the control group. Among children with 16S + 23S rRNA (+) in the dental biofilm and saliva, BANA test positive results were found higher than children with 16S + 23S rRNA (−) in their oral cavities. BANA test detects *Treponema denticola, Porphyromonas gingivalis*, and *Tannerella forsythia* present on the dorsum of the tongue. The reason for this result might be an increase in the oral prevalence of these volatile sulfur compounds producing odorous periodontopathic microorganisms in the oral cavity with *H.pylori* colonization. Our study is the first study in which BANA test was used to investigate the relationship between halitosis and the presence of *H.pylori* in the oral cavity.

### Oral parameters

Our plaque index scores of children who were 16S + 23S rRNA (+) in dental biofilm and gingival index scores of children who were 16S + 23S rRNA (−) in saliva were found significantly higher than children who were 16S + 23S rRNA (−) in oral samples. These results were found in accordance with the result of Liu et al. [25], Gürbüz et al. [35], Butt et al. [36], Song et al. [81] and Liu et al. [6], but found different from the studies of Berroteran et al. [37], Al-Refai et al. [26] and Nguyen et al. [39]. Dental biofilm provides an optimal pH, temperature and microaerophilic environment required for the survival of *H.pylori* [24]. After systemic antibiotic therapy, gastric *H. pylori* can be eradicated from the stomach, but not from the dental biofilm. We believe that could lead to re-infection and treatment failure due to the continued presence of *H.pylori* in the dental biofilm. Presence of the bacterium in dental biofilm and saliva may play a role in the transmission of bacterial infection from one person to another. We, therefore, believe that regular professional dental cleaning is required to prevent an increase in plaque load and the number of bacteria in the oral cavity in order to prevent gastric colonization.

Kolho et al. [82] did not find any relationship between the gastric presence and dental caries. Liu et al. [6] found a relationship, on the other hand, Berroteran et al. [37] and Banatvala et al. [83] did not find any relationship between the oral presence and dental caries. In this study, some differences in DMFT/S and dmft/s scores were determined among groups*.* But no difference was found in *S.mutans* and *Lactobacilli* numbers. This was not sufficiently significant to support the assumption that a relationship presents between oral and gastric colonization of *H.pylori* and dental caries. Our results were found in accordance with the results of Berroteran et al. [37], Banatvala et al. [83] and Kolho et al. [82] but not in accordance with the results of Liu et al. [6].

Vahadi et al. [57] reported that in cases where salivary flow rate decreases, the accumulation of plaque in the oral cavity increases because of reduced cleansing properties of saliva. In this study, there were no relationship found between the salivary flow rate and the gastric/oral presence of *H.pylori*. The oral acidic pH is suggested to represent a favorable microenvironment for *H.pylori* despite the presence of abundant bacterial flora [76]. Torcatoru et al. [21] reported that the area around the dental biofilm has low reduction-oxidation potential, which promotes the growth of facultative anaerobes. The acidic pH reduced due to the fermentation process of carbohydrates which is ideal for *H.pylori* growth. In the present study, a clear relationship was found between the pH levels of saliva and the presence of *H.pylori* in the dental biofilm and saliva samples. If the decrease in pH in the oral cavity can be prevented with good oral hygiene, diet, and regular plaque control, we believe that the settlement and growth of *H.pylori* in the oral cavity can be prevented. Good oral hygiene not only prevents gingivitis it may also help secondarily reduce the risk of stomach infection or reinfection with *H.pylori*. In the current study, no relationship was found between the gastric colonization [30, 39, 41, 55, 63] and the oral colonization [4, 39, 42, 55–57, 83] with oral hygiene practices. However, Liu et al. [6, 25] showed a relationship between the oral colonization and oral hygiene habits. It was not possible to examine the effectiveness of toothbrushing in the evaluation of oral hygiene practices among participants. Subjective data taken from children and parents might not reflect the real success of brushing.

### Demographic Parameters

No relationship was found between the gastric presence and variables like family income, education status, and family population. Our study was found in accordance with the results of Anand et al. [31] and Altındiş et al. [55] and not in accordance with Ahmed et al. [12], Nahar et al. [32], Wichelhaus et al. [57], and Ghosh et al. [27].

### Strength and limitations

In this study, the RT- PCR method was used which has higher sensitivity and specificity in detection of *H.pylori* than other methods [84]. *Helicobacter pylori* is mainly acquired in childhood thus this study was conducted on a group of children aged between 5–16. In the literature, few studies [5, 72] were found using RT-PCR system in detecting *H.pylori* in the oral cavities of children. Also in this study, two genes of the bacterium were simultaneously amplified for the detection of the organism in both dental biofilm and saliva samples which were more reliable when compared to one gene amplification only [61]. The limitation of the study is the lack of the investigation of serotype similarity of *H.pylori* in dental biofilm, saliva, and gastric samples. Also, the control group consisted of healthy children without any systemic diseases. Therefore no biopsies were taken from them. In our study, convenience sampling was used which is a type of non-probability or non-random sampling where members of the target population that meet certain practical criteria, such as easy accessibility, geographical proximity, availability at a given time, or the willingness to participation [85]. Long-term follow-up of the children’s oral health and additional research of *H.pylori* in the oral cavity of the children involved in the study, after eradication therapy also has vital importance.

## Conclusions

*Helicobacter pylori* can occur in the oral cavity aside and independently from the stomach. However, the number of bacteria was found high in the oral cavities (in dental biofilm and saliva) of children with gastric *H.pylori*. An association between oral presence of *H.pylori* and halitosis, DMFS and pH was also found. Thus, reinfection and treatment failure may continue due to the presence of *H.pylori* in the oral cavity. The rate of recurrence can be reduced by long-term professional dental plaque control and improving the oral health status, thus a viable bacterial count which is required for successful infection of the gastric mucosa maintained too low. So the small number existing at this level might not enough to infect gastric mucosa after passing into the stomach with saliva or swallowed food.

## Additional file

Additional file 1: Questionnaire: was directed to the children’s parents and used to assess the different factors associated with the study. (PDF 197 kb)

## Abbreviations

RT-PCR: real-time polymerase chain reaction
NSAIDs: nonsteroidal anti-inflammatory drugs
BANA test: benzoyl-DL- arginine-naphthylamide test
CLO test: Campylobacter-like organism test
DMFT/S: decayed, missing and filled permanent teeth/surfaces
dmft/s: decayed, missing, and filled primary teeth/surfaces
