# Supplementary material for: The investigation of Helicobacter pylori in the dental biofilm and saliva samples of children with dyspeptic complaints
Source: BMC Oral Health. 2017 Mar 21;17:67. doi: 10.1186/s12903-017-0361-x (PMC5361728; doi:10.1186/s12903-017-0361-x)
Supplement: Additional file 1: — Questionnaire: was directed to the children’s parents and used to assess the different factors associated with the study. (PDF 197 kb) [file 12903_2017_361_MOESM1_ESM.pdf]

Additional File 1: Questionnaire

Date :
Protocol number :
Birth Date : ____/_____/_______

Gender : Female ☐ Male ☐

1. Education level of the mother and the father: Illiterate ☐ Primary School ☐ High School ☐ University ☐
2. Total income of the family per month: Below minimum wage ☐ Minimum wage ☐ Between 2000-5000 ☐ Above 5000₺ ☐
3. The number of family members living together: 2 ☐ 3 ☐ 4 ☐ 5 ☐ 6 ☐ Above 6 ☐
4. Pets at home: Yes ☐ No ☐
5. Any medical history except dyspeptic complaints: Yes ☐ __________ No ☐
6. Regularly used medicines: Yes ☐ __________________ No ☐
7. Dyspeptic complaints: Epigastric pain ☐ Nausea ☐ Diarrhea ☐ Constipation ☐ Reflux ☐ Vomiting/ Regurgitation ☐
8. Daily toothbrushing frequency of the child : Once daily ☐ Twice daily ☐ Non-regular ☐ None ☐
9. Frequency of toothbrush replacing: Less than 3 months ☐ 3-6 months ☐ 6-12 months ☐ Over a year ☐
10. Sort of used toothbrush: Soft ☐ Medium ☐ Hard ☐
11. Usage of dental products: Toothpaste ☐ Mouthrinse ☐ Dental Floss ☐
12. Feeding with premasticated food: Yes ☐ No ☐
13. Common usage of glasses and spoons: Yes ☐ No ☐
14. Sugar consumption frequency: Several times a week ☐
    1-2 times daily ☐
    3 or more times daily ☐
    No consumption ☐
15. Beverage preference: Water ☐ Milk ☐ Fruit juice ☐ Tea ☐ Cola ☐
